# Capsaicin for Rhinitis

**DOI:** 10.1007/s11882-016-0638-1

**Published:** 2016-08-03

**Authors:** Wytske Fokkens, Peter Hellings, Christine Segboer

**Affiliations:** Department of Otorhinolaryngology, Academic Medical Centre, Amsterdam, The Netherlands

**Keywords:** Capsaicin, Rhinitis, Idiopatic rhinitis, Non-allergic rhinitis

## Abstract

Rhinitis is a multifactorial disease characterized by symptoms of sneezing, rhinorrhea, postnasal drip, and nasal congestion. Non-allergic rhinitis is characterized by rhinitis symptoms without systemic sensitization of infectious etiology. Based on endotypes, we can categorize non-allergic rhinitis into an inflammatory endotype with usually eosinophilic inflammation encompassing at least NARES and LAR and part of the drug induced rhinitis (e.g., aspirin intolerance) and a neurogenic endotype encompassing idiopathic rhinitis, gustatory rhinitis, and rhinitis of the elderly. Patients with idiopathic rhinitis have a higher baseline TRPV1 expression in the nasal mucosa than healthy controls. Capsaicin (8-methyl-N-vanillyl-6-nonenamide) is the active component of chili peppers, plants of the genus Capsicum. Capsaicin is unique among naturally occurring irritant compounds because the initial neuronal excitation evoked by it is followed by a long-lasting refractory period, during which the previously excited neurons are no longer responsive to a broad range of stimuli. Patients with idiopathic rhinitis benefit from intranasal treatment with capsaicin. Expression of TRPV1 is reduced in patients with idiopathic rhinitis after capsaicin treatment. Recently, in a Cochrane review, the effectiveness of capsaicin in the management of idiopathic rhinitis was evaluated and the authors concluded that given that many other options do not work well in non-allergic rhinitis, capsaicin is a reasonable option to try under physician supervision. Capsaicin has not been shown to be effective in allergic rhinitis nor in other forms of non-allergic rhinitis like the inflammatory endotypes or other neurogenic endotypes like rhinitis of the elderly or smoking induced rhinitis.

## Introduction

Rhinitis is a multifactorial disease characterized symptoms of sneezing, rhinorrhea, postnasal drip, and nasal congestion. Rhinitis affects 10–40 % of the population in the industrialized countries, which is responsible for billions of dollars of healthcare expenditure and impairs the quality of life those affected [1•]. Rhinitis constitutes a risk factor for asthma and is associated with chronic conditions such as rhinosinusitis [2••]. Classification of rhinitis entities on the basis of phenotypes has facilitated their characterization and has helped practicing clinicians to efficiently approach rhinitis patients [2••]. Allergic rhinitis (AR) is defined as a symptomatic disorder of the nose induced by an IgE-mediated inflammation after allergen exposure of the membranes lining the nose. Non-allergic rhinitis (NAR) is characterized by rhinitis symptoms without systemic sentization of infectious etiology. A number of different phenotypes exists of which the most important are idiopathic rhinitis and irritative-induced rhinitis, like smokers rhinitis, drug-induced rhinitis, gustatory rhinitis, non-allergic rhinitis with eosinophilia syndrome (NARES), atrophic rhinitis, rhinitis of the elderly, and hormonally induced rhinitis [2••, 3]. Some of these phenotypes, like rhinitis of the elderly, have very clear characteristics: watery rhinorrhea without other significant rhinitis symptoms, but other forms are not so easy to discriminate and sometimes also overlap. In between NAR and AR is another disease called local allergic rhinitis (LAR) that is characterized by local IgE without systemic sensitization but with allergen-induced symptoms. Although there are some data pointing to about half of the rhinitis patients having NAR, the prevalence of the various phenotypes of NAR is largely unknown. To make things more complicated, patients may have a phenotype called mixed rhinitis with components of NAR and AR, e.g., a patient with seasonal allergic symptoms based on AR but perennial non-allergic symptoms. Finally, the so-called hyperreactivity is an increased sensitivity of the nasal mucosa to various non-specific stimuli. Both allergic rhinitis (AR) and non-allergic rhinitis (NAR) patients can elicit nasal hyperreactivity symptoms, and no quantitative or qualitative differences in nasal hyperreactivity can be found between AR and NAR patients [4•]. Moreover, it is not possible to differentiate NAR subpopulations based on physical or chemical stimuli [4•].

When we try to define NAR based on endotypes, we might say that at least there is an inflammatory endotype with usually eosinophilic inflammation encompassing at least NARES and LAR and part of the drug induced rhinitis (e.g., aspirin intolerance) [5, 6••]. Another clear endotype is the neurogenic endotype encompassing idiopathic rhinitis, gustatory rhinitis, and rhinitis of the elderly and other phenotypes with a strong neurological component [6••].

### Diagnosing NAR

NAR is clinically usually diagnosed based on a thorough history and a SPT to rule out AR.

In epidemiological research, it is possible to rule out AR with the proper questions in a fairly accurate way [7]. In clinical research, NAR is diagnosed by a positive reaction to CDA [8, 9]. Histamine, methacholine, and capsaicin have been shown not to be able to discriminate NAR patients from healthy controls [3]. A PNIF meter can be provided to take home in case patients complain of hyperreactivity attacks with nasal obstruction [10, 11]. Many patients with NAR have uncontrolled disease [12].

### Pathophysiology of Neurogenic Rhinitis Endotype

The neural regulation of the upper airways is complex and consists of a number of interacting nervous systems. Sensory, parasympathetic, and sympathetic nerves regulate epithelial, vascular, and glandular processes in the nasal mucosa.

Sensory nerves originating form ethmoidal and nasopalatine branches of the trigeminal nerve transmit afferent sensory input from the nasal epithelium, blood vessels, and secretory glands. These nerve endings extend close to the nasal epithelial surface and between tight junctions of epithelial cells. These nerve fibers respond to environmental irritants such as cigarette smoke, pain, and variations in temperature and osmolarity and thus play an important role in NAR. Activation of nasal afferent nerves by aspecific factors results in defensive efferent responses, such as sneezing, and glandular and/or vascular activation, leading to rhinorrhea and nasal congestion. Both transient receptor potential ankyrin 1 (TRPA1) and vanilloid 1 (TRPV1) receptors are abundantly expressed in these C-fiber sensory nerves and can be activated by a number of endogenous inflammatory mediators [6••, 13•]. C fibers are often defined as falling within two broad categories, peptidergic and non-peptidergic. The peptidergic C fibers can also locally release neuropeptides (antidromic pathway) such as substance P or calcitonin gene related-peptide (CGRP) after stimulation of the TRP receptors. This neuropeptides release gives rise to vasodilation, extravasation and hypersecretion resulting in symptoms of rhinitis. Patients with idiopathic rhinitis compared to healthy controls have been shown to overexpress TRPV1 in the nasal mucosa, and they have increased SP levels in nasal secretions [14••].

Apart from the potential dysregulation in the sensory nerves, an imbalance of the efferent nasal reflex arc of the autonomic nerve supply to the nasal plays a role in some forms of NAR. Equal contributions from both components maintain a balance between the vasoconstriction and vasodilation of nasal vasculature and stimulation of serous glands. An imbalance of these components leads to glandular hypersecretion and plays an important role, e.g., in rhinitis of the elderly. The anticholinergic agent ipratropium bromide is a highly effective treatment for hypersecretion in idiopathic rhinitis [15, 16] but does not have any influence on other symptoms like nasal blockage or sneezing [17, 18].

### Capsaicin

Capsaicin (8-methyl-*N*-vanillyl-6-nonenamide) is the active component of chili peppers, plants of the genus Capsicum. Along with other related compounds, it belongs to a group of chemicals identified as capsaicinoids. Capsaicin produces a burning sensation when a tissue comes into contact with it. This occurs via binding to transient receptor potential vanilloid 1 (TRPV1) receptor, an ion channel-type receptor, which can be stimulated by heat and physical abrasion. Capsaicin is unique among naturally occurring irritant compounds because the initial neuronal excitation evoked by it is followed by a long-lasting refractory period, during which the previously excited neurons are no longer responsive to a broad range of stimuli. This process known as defunctionalization has been exploited for therapeutic use of capsaicin in various painful conditions [19]. A beneficial role of capsaicin has been reported in obesity, cardiovascular and gastrointestinal conditions, various cancers, neurogenic bladder, and dermatologic conditions [19]. Recent studies focusing on tumor immunity, allergy, and inflammation have noted the immunotherapeutic effects of capsaicin [20–23]. In a large prospective study of over 0.5 million adults from ten geographically diverse areas across China, the habitual consumption of spicy food was found to be inversely related with total and specific mortality [24].

When sprayed in the nose, capsaicin induces burning, rhinorrhea, nasal blockage, and lacrimation. TRPV1 activation leads to a cationic influx in the nerve terminals, resulting in neuronal excitation, an increase in intracellular Ca2+ concentration, and antidromic release of neuropeptides, which potentially triggers a local inflammatory response. The therapeutic effect of intranasal capsaicin application is thought to be induced by the abovementioned defunctionalization and/or degeneration of nerve terminals by the massive Ca2+ influx. For unclear reasons, most patients with IR only experience the recurrence of nasal symptoms after several months and subsequently seek the reapplication of capsaicin. In a study comparing patients with idiopathic rhinitis and controls, van Gerven et al. reported that patients with IR had higher baseline transient receptor potential cation channel subfamily V, receptor 1 (TRPV1) expression in the nasal mucosa, and higher concentrations of substance P (SP) in nasal secretions than healthy controls. Expression of TRPV1, transient receptor potential cation channel subfamily M, receptor 8 (TRPM8), and protein gene product 9.5 (PGP 9.5) was reduced in patients with IR after capsaicin treatment. Capsaicin did not alter the mast cell marker c-KIT or nasal epithelial morphology nor did it induce apoptosis or necrosis in cultured human nasal epithelial cells and mast cells [14••]. Azelastine has also been found to be clinically effective in the treatment of NAR, but its mechanism(s) of action is still poorly elucidated. Using in vitro cell line mice neuronal cells as surrogates for submucosal sensory neurons, and human nasal epithelial cells (hNEC), Singh et al. showed that azelastine, similar to capsaicin, exhibits direct activity on TRPV1 ion channels that may represent a novel mechanistic pathway explaining its clinical efficacy in NAR [25•]. Also, a recent study has determined the inhibition of TRPV1 but not TRPA1 channels by tiotropium and ipratropium [26•]. These studies suggest that symptoms mediated by neuronal TRPV1 are inhibited by tiotropium via mechanisms distinct from its anticholinergic action [6••, 26•].

### Capsaicin in AR

One small trial did not find evidence that intranasal capsaicin had a therapeutic effect in allergic rhinitis [27•]. A small pharmacological effect on clinical histamine dose response was found. After treatment, leukotriene levels in nasal lavage did not increase in the capsaicin group [27•]. Also, a TRPV1 blocker had no effect on total symptom score, PNIF and ECP levels in allergen-challenged patients with seasonal allergic rhinitis. The individual symptoms, nasal itch or sneezes, were also not affected [28]. These findings may indicate that TRPV1 is not a key mediator of the symptoms in allergic rhinitis. However, additional studies, using drug formulations with a prolonged duration of action, should be conducted before TRPV1 is ruled out as a drug target in allergic rhinitis [28].

### Capsaicin in NAR

Patients with idiopathic rhinitis benefit from intranasal treatment with capsaicin (i.e., the main pungent agent in hot chili peppers). In 1997, Blom et al. published the first placebo-controlled trial showing therapeutic efficacy [29, 30].

Recently, in a Cochrane review, the effectiveness of capsaicin in the management of idiopathic rhinitis was evaluated [31••]. Overall, the quality of the evidence was judged to be of low to moderate quality. The authors concluded that given that many other options do not work well in non-allergic rhinitis, capsaicin is a reasonable option to try under physician supervision.

Four studies (five publications) involving 302 adult patients with moderate-severe idiopathic rhinitis were analyzed. Two studies compared capsaicin to placebo [29, 30, 32]; one study compared two treatment regimens of capsaicin: five treatments in 1 day versus five treatments given every 2 to 3 days during 2 weeks [33]. The study by Ciabatti, in addition to comparing capsaicin with placebo, also compared three different doses of capsaicin to each other [32]. Finally, Havas et al. compared capsaicin with budesonide [34].

The studies in the Cochrane review had follow-up periods ranging from 4 to 38 weeks [31••]. Of the two studies that compared capsaicin with placebo, one study reported that capsaicin resulted in an improvement of overall nasal symptoms (a primary outcome) measured on a visual analogue scale (VAS) of 0 to 10 [29, 30]. There was a mean difference (MD) of −3.34 (95 % confidence interval (CI) −5.24 to −1.44), MD −3.73 (95 % CI −5.45 to −2.01) and MD −3.52 (95 % CI −5.55 to −1.48) at 2, 12, and 36 weeks posttreatment, respectively. The other study reported that, compared to placebo, capsaicin (at 4 μg/puff) was more likely to produce overall symptom resolution (reduction in nasal blockage, sneezing/itching/coughing, and nasal secretion measured with a daily record chart) at 4 weeks posttreatment (a primary outcome) [32]. The risk ratio (RR) was 3.17 (95 % CI 1.38 to 7.29). In the study that compared capsaicin to budesonide, it was shown that patients treated with capsaicin had a better overall symptom score compared to those treated with budesonide (MD 2.50, 95 % CI 1.06 to 3.94, VAS of 0 to 10) [34]. The last study compared two different regimens of capsaicin administration: five treatments in 1 day versus five treatments given every 2 to 3 days during 2 weeks [33]. Using daily record charts, the study showed comparable results whether treatment was given as five treatments every other 2–3 days or five times in 1 day with hours between the applications.

One study measured the levels of leukotrienes C4/D4/E4, prostaglandin D2, and tryptase in the nasal lavage and the expression of CD1, CD3, CD25, CD68, IgE, MBP (i.e., BMK13 antibody), chymase, tryptase, synaptophysin, and neurofilament and found no statistically significant differences between the groups [29, 30]. Capsaicin treatment has been shown to be safe: blood pressure, heart rate, olfactory function, and mucosal sensibility are not affected by the treatment [33]. The treatment effect lasts at least 1 year and can easily be repeated [3].

In the Cochrane review, one randomized, placebo-controlled, double-blind, parallel study of 21 days total duration (7 days pre-treatment and 14 days during treatment), which compared Sinus Buster (a homeopathic preparation of Capsicum annuum and Eucalyptol) with placebo (filtered water) was excluded because the dosage of Capsaicin was unclear [31••, 35]. The participants were patients with non-allergic rhinitis and mixed rhinitis. Results of this study showed improvement in total nasal symptom score, nasal pain, headache, and sinus pressure compared with placebo with a self-reported quick onset of action (less than a minute) [35].

## Conclusions

To our knowledge, there are no studies available to evaluate the effect of capsaicin in other forms of NAR. In the experience of the author of this paper, capsaicin is not effective in rhinitis of the elderly or in NAR caused by smoking.

For now, capsaicin nasal spray, with adequate concentration, is not produced commercially and thus needs some attention to be produced by the (hospital) pharmacist. The now available commercial products have unknown concentrations of capsaicin but may be also effective [35]. New drugs targeting TRPV1 are developed [36] and may offer the potential to control medical conditions characterized by sensory neuronal hyper-responsiveness, including the nasal hyper-reactivity that underlies NAR [37].
